# Diffusion-weighted imaging and apparent diffusion coefficient values for evaluating terminal ileitis in patients with Crohn’s disease

**DOI:** 10.1590/0100-3984.2019.0011

**Published:** 2019

**Authors:** Evandra Durayski, Guilherme Watte, Gabriel Sartori Pacini, Diego Hermindo Roman, Marta Brenner Machado, Edson Marchiori, Bruno Hochhegger, Matteo Baldisserotto

**Affiliations:** 1 School of Medicine, Graduate Program in Medicine and Health Sciences, Pontifical Catholic University of Rio Grande do Sul (PUCRS), Porto Alegre, RS, Brazil.; 2 Medical Imaging Research Lab (Labimed), Department of Radiology, Pavilhão Pereira Filho Hospital, Irmandade Santa Casa de Misericórdia de Porto Alegre, Porto Alegre, RS, Brazil.; 3 Federal University of Rio de Janeiro (UFRJ), Rio de Janeiro, RJ, Brazil.

**Keywords:** Crohn’s disease, Diffusion magnetic resonance imaging, Inflammation, Ileitis, Intestine, small, Magnetic resonance imaging

## Abstract

**Objective:**

To determine the accuracy of diffusion-weighted imaging (DWI) in identifying terminal ileitis in patients with Crohn’s disease.

**Materials and Methods:**

This was a retrospective study of 38 consecutive patients with Crohn’s disease who underwent magnetic resonance enterography with DWI in a 3.0 T scanner. The patients were divided into two groups, on the basis of colonoscopy and biopsy findings: active inflammation; and inactive disease. Apparent diffusion coefficient (ADC) values were determined, as were the magnetic resonance index of activity (MaRIA) and the Clermont score.

**Results:**

Of the 38 patients evaluated, 18 (47%) had active inflammation. The patients with active inflammation showed greater restricted diffusion, more pronounced mucosal edema, greater wall thickening, a higher MaRIA, and a higher Clermont score than did those with inactive disease. The level of interobserver agreement (intraclass correlation coefficient) was excellent for the MaRIA and the Clermont score, whereas it was substantial for the ADC values. For identifying colonoscopy-proven inflammation, the best ADC cut-off point was 2.1 × 10^−3^ mm^2^/s, which had a sensitivity of 88.8% and a specificity of 95.0%, whereas DWI presented an overall accuracy of 89.4%, with a sensitivity of 88.9% and a specificity of 90.0%.

**Conclusion:**

Visual analysis of the DWI sequence has good accuracy in detecting terminal ileitis in patients with Crohn’s disease. In addition, low ADC values have good sensitivity for detecting colonoscopy-proven inflammation.

## INTRODUCTION

Crohn’s disease is an idiopathic inflammatory bowel disease that primarily affects the terminal ileum. The diagnosis of Crohn’s disease is established by the combined analysis of clinical, endoscopic, radiological, laboratory, and histological data^(1,2)^. The course of the disease is markedly episodic, with periods of exacerbation and remission^(3)^.

Although colonoscopy is the gold-standard method for evaluating Crohn’s disease, magnetic resonance enterography (MRE) has become a promising alternative to colonoscopy in the monitoring of patients with Crohn’s disease^(4,5)^. Diffusion-weighted imaging (DWI) sequences for MRE do not use intravenous contrast agents and allow the evaluation of the movement (Brownian motion) of water molecules in tissues. Analysis of the diffusion sequence can be performed in two ways: visual analysis, to identify restricted diffusion; and quantitative analysis, in the form of apparent diffusion coefficient (ADC) values. Studies have shown that restricted diffusion in the wall of the intestine correlates with inflammation. Therefore, diffusion has the potential to be a biomarker of intestinal inflammation in Crohn’s disease^(3)^.

To date, there have been few studies evaluating the potential of ADC values to identify inflammation^(6)^. In addition, such studies have involved small numbers of patients and have not correlated their results with pathology^(7)^. Furthermore, new imaging techniques have been developed. Therefore, additional studies are warranted. The present study aims to determine the accuracy of DWI in identifying signs of terminal ileitis in patients with Crohn’s disease.

## MATERIALS AND METHODS

### Study population

This was a retrospective observational study of patients treated in the Department of Gastroenterology of São Lucas Hospital, in the city of Porto Alegre, Brazil, between March 2014 and September 2017. We selected patients with Crohn’s disease who underwent colonoscopy and biopsy of the terminal ileum up to one month prior to undergoing MRE. The present study was approved by the medical research ethics committee of our institution.

Two groups were defined according to the results of the colonoscopy and subsequent biopsy, which is considered the gold standard^(8)^: active inflammation; and inactive disease. The active inflammation group consisted of patients diagnosed with Crohn’s disease who underwent colonoscopy and a subsequent biopsy that showed evidence of acute inflammation in the terminal ileum during a period of disease activity. The inactive disease group consisted of patients diagnosed with Crohn’s disease who underwent colonoscopy and a subsequent biopsy that showed no signs of acute inflammation in the terminal ileum (i.e., those with clinically controlled disease or those who were in a period of remission). The exclusion criteria were incomplete MRE data and technical difficulties.

### MRE technique

On the day of the MRE, patients fasted for at least 8 h before the examination. The images were acquired in a 3.0 T scanner (Signa HDxt; General Electric, Milwaukee, WI, USA). The patients were placed in the supine (most comfortable) position. The various sequences were acquired through the abdomen and pelvis using an 8-channel body coil. To reduce intestinal peristalsis and achieve adequate distention of the terminal ileum, one ampule of scopolamine (Buscopan; Boehringer Ingelheim, Ingelheim, Germany) was administered to each patient. When appropriate, a gadolinium-based contrast agent was administered. Subsequently, the sequences described below were acquired and their respective parameters were determined (Table 1).

**Table 1:** Characteristics of the MRE sequences used in the study protocol.

Sequence	Plane	FOV (cm)	TE (ms)	TR (ms)	Flip angle (º)	Slice thickness (mm)	Acquisition time (s)
T2-weighted 2D							
SSFSE	Coronal	45	100.0	1363.0	90	5	33
FS SSFSE	Coronal	45	80.0	1330.0	90	5	16
2D FIESTA	Coronal	42	2.0	4.5	50	4	16
T2-weighted SSFSE	Axial	45	100.0	1064.0	90	7	18
T1-weighted 2D							
SPGR	Axial	41	7.7	50.0	12	8	112
SPGR MT	Axial	41	7.7	50.0	12	8	112
T1-weighted 3D LAVA FS	Coronal	44	2.2	4.6	12	5	17
70 s	Coronal	44	2.2	4.6	12	5	17
420 s	Coronal	44	2.2	4.6	12	5	17
T1-weighted 3D LAVA FS	Axial	44	2.2	4.6	12	5	17
DWI (b-values of 0, 50, 400, and 800)	Axial	38	60.7	7500.0	90	8	218

FOV, field of view; TE, echo time; TR, repetition time; 2D, two-dimensional; FS, fat-saturated; SSFSE, single-shot fast spin-echo; FIESTA, fast imaging employing steady-state acquisition with fat suppression; SPGR, spoiled gradient-recalled; MT, magnetization transfer; LAVA, liver acquisition with volume acceleration.

### Image analysis

Image postprocessing was performed on a dedicated workstation (ADW; GE Medical Systems, Milwaukee, WI, USA). All images were interpreted by two radiologists, each with 10 years of experience, who were blinded to the clinical results and worked independently. Disagreements between the radiologists were resolved by consensus; if a consensus could not be reached, a third radiologist, with 30 years of experience, made the final decision.

The following variables were analyzed (Table 2): restricted diffusion; ADC; wall thickening in the intestinal loop; mucosal edema; ulcerations; stricture; relative contrast enhancement (RCE); magnetic resonance index of activity (MaRIA); and Clermont score. A dichotomous qualitative analysis was performed with a b-value of 800 s/mm^2^ in the diffusion sequence^(9)^ of the wall of the ileum. When there was high signal intensity in the DWI sequence and low signal intensity on the ADC map, the patient was classified as having restricted diffusion. A quantitative evaluation of the ADC values was also performed as described in the study conducted by Hordonneau et al.^(8)^. A 10-30 mm^2^ region of interest (ROI) was drawn over the area with the highest signal intensity in the bowel wall. Thickening of the wall of the ileum was measured in millimeters at its thickest point or at the site with the most severe inflammation^(10)^. As a criterion, the thickest portion of the most distended segment or the site of the most severe inflammation (> 3 mm) was measured^(9)^.

**Table 2:** Parameters of interest for the MRE assessment of bowel inflammation in Crohn’s disease and corresponding MRE findings.

MRE parameter	Definition/cut-off value
DWI	Hyperintense signal at a b-value of 800 s/mm
Mean ADC	ROI at 10-30 mm^2^
Wall thickening	≥ 3 mm
Intramural edema	Hyperintense signal on fat-saturated T2-weighted images
Ulcerations	Appear as small focal breaks in the intraluminal surface of the bowel
Stricture	Upstream lumen > 3 cm
Hyperenhancement	Attenuation on a contrast-enhanced scan higher in a segment that is not contracted than in nearby small bowel segments that are normal
MaRIA	≥ 11
Clermont score	≥ 12.5

When the signal seen on the T2-weighted image was hyperintense in relation to that of the psoas muscle signal, the examination was considered positive for intramural edema^(3,9)^. Ulcerations were defined as small focal losses of signal continuity on the intraluminal surface of the intestinal wall, containing air or enteric contrast media. Stricture was defined as luminal narrowing in the area of Crohn’s disease with unequivocal upstream dilatation > 3 cm^(10)^. When the attenuation intensity on the contrast-enhanced scan was greater in the segment that was not contracted than in the nearby normal segments of the small bowel, the examination was considered positive for segmental mural hyperenhancement.

The RCE was calculated by applying the following formula^(9)^:

$$\begin{array}{c} \mathrm{RCE}=\left[\left(\mathrm{post}-\mathrm{gadolinium}\;\mathrm{WSI}-\mathrm{pre}-\mathrm{gadolinium}\;\mathrm{WSI}\right)\right.\\ /\left.\left(\mathrm{pre}-\mathrm{gadolinium}\;\mathrm{WSI}\right)\right]\times 100\times \left(\mathrm{pre}-\mathrm{gadolinium}\;\mathrm{SD}\right.\\ \left.\mathrm{noise}/\mathrm{post}-\mathrm{gadolinium}\;\mathrm{SD}\;\mathrm{noise}\right) \end{array}$$

where *WSI* is the wall signal intensity and *SD* is the standard deviation. The MaRIA was calculated with the following formula^(4,9,10)^:

$$\begin{array}{c} \mathrm{MaRIA}=1.5\times \mathrm{wall}\;\mathrm{thickening}\left(\mathrm{mm}\right)+0.02\times \mathrm{RCE}+\\ 5\times \mathrm{edema}+10\times \mathrm{ulceration} \end{array}$$

The Clermont score was also calculated, by using the following formula^(9)^:

$$\begin{array}{c} 1.646\times \mathrm{int}\mathrm{estinal}\;\mathrm{wal}\;\mathrm{thickening}-1.321\times \mathrm{ADC}+\\ 5.613\times \mathrm{edema}+8.306\times \mathrm{ulceration}+5.039 \end{array}$$

### Colonoscopy and biopsy

Colonoscopies were performed after bowel preparation with 4 L of polyethylene glycol. An experienced endoscopist performed all of the examinations, during which biopsy specimens were collected. Visualization of inflamed mucosa during the colonoscopy or evidence of bowel inflammation in the biopsy specimen was accepted as proof of inflammation and noted for each segment. The colonoscopy findings were evaluated in accordance with the Crohn’s disease endoscopic index of severity^(11)^. A pathologist with 15 years of experience assessed all biopsy samples.

### Statistical analysis

Data are presented as absolute and relative frequencies (for categorical variables) or as means and standard deviations (for continuous variables). To evaluate associations between variables, we used Fisher’s exact test or chi-square test. To compare continuous variables, Student’s t-tests or unequal variance t-tests were used. Spearman’s rank correlation coefficient was used in order to assess the linear associations between continuous variables.

Interobserver agreement was assessed by calculating the intraclass correlation coefficient (ICC) for continuous variables and the kappa statistic (κ) for categorical variables. The κ values were interpreted as follows^(12)^: poor (< 0.01); slight (0.01-0.20); fair (0.21-0.40); moderate (0.41-0.60); substantial (0.61-0.80); and almost perfect (0.81-1.00). The ICC values were interpreted as follows^(13)^: poor (< 0.40); fair (0.40-0.59); good (0.60-0.74); and excellent (≥ 0.75).

Logistic regression models were constructed to obtain a classification for each case (inflammation). The Hosmer-Lemeshow goodness-of-fit test was conducted with a subsequent logistic regression classifier fitting to the selected features. To assess the performance of each logistic regression classifier, the threshold analysis was performed by constructing receiver operating characteristic (ROC) curves and calculating the areas under the curves (AUCs), after which we calculated Youden’s index [1 − (sensitivity + specificity)] and efficiency [defined as the probability that the test and diagnosis agree: (*true positive* + *true negative*) ∕ *total*]. The sensitivity, specificity, and overall accuracy were assessed by logistic analysis.

In all cases, values of *p* < 0.05 were considered statistically significant. Statistical analyses were performed using the Predictive Analytics Software package, version 18.0 (SPSS Inc., Chicago, IL, USA) and Stata software, version 11.0 (StataCorp, College Station, TX, USA).

## RESULTS

The characteristics of the study subjects are described in Table 3. In total, 38 patients with Crohn’s disease were included in the study. The sample comprised equal numbers of male and female subjects, and the mean age was 36 ± 14 years. The mean time from biopsy to MRE was 14 ± 8.9 days. Of the 38 patients evaluated, 18 (47%) had active inflammation.

**Table 3:** Baseline characteristics of the patients with Crohn’s disease.

Characteristic	(N = 38)
Male, n (%)	19 (50)
Age (years), mean ± SD	36 ± 14
Disease duration (years), mean ± SD	7 ± 5
Inflammation on colonoscopy, n (%)	18 (47)
Wall thickening (mm), mean ± SD	4.32 ± 2.38
Intramural edema, n (%)	12 (32)
Ulcerations, n (%)	3 (8)
Stricture, n (%)	4 (10)
ADC (10^-3^ mm^2^/s), mean ± SD	2.38 ± 1.07
RCE (%), mean ± SD	108.52 ± 48.24
MaRIA, mean ± SD	11.0 ± 6.10
Clermont score, mean ± SD	11.4 ± 7.20

The patients who presented with active terminal ileitis, as identified by colonoscopy and biopsy, showed a more intense signal on DWI, more pronounced mucosal edema on the T2-weighted image, greater wall thickening, lower ADC values, higher MaRIAs, and higher Clermont scores than did those with inactive disease. The main results of this analysis are summarized in Table 4.

**Table 4:** Comparisons between the patients with and without active terminal ileitis.

Variable	Active inflammation		
	No (n = 20)	Yes (n = 18)	*p*
Age (years), mean ± SD	35.5 ± 13.4	35.7 ± 14.4	0.781
Male, n (%)	10 (50.0)	9 (50.0)	1.0
Restricted diffusion, n (%)	2 (5.3)	16 (42.1)	< 0.001
Edema, n (%)	2 (5.3)	10 (26.3)	0.004
Ulcerations, n (%)	0	3 (7.9)	0.097
Stricture, n (%)	0	4 (10.0)	0.007
Wall thickening, mean ± SD	2.84 ± 0.83	5.97 ± 2.47	< 0.001
ADC (10^-3^ mm^2^/s), mean ± SD	3.04 ± 0.95	1.65 ± 0.65	< 0.001
RCE (%), mean ± SD	101.14 ± 51.46	116.72 ± 44.38	0.327
MaRIA, mean ± SD	6.78 ± 2.50	15.74 ± 5.47	< 0.001
Clermont score, mean ± SD	6.33 ± 3.45	17.19 ± 5.99	< 0.001

**Figure 1:**
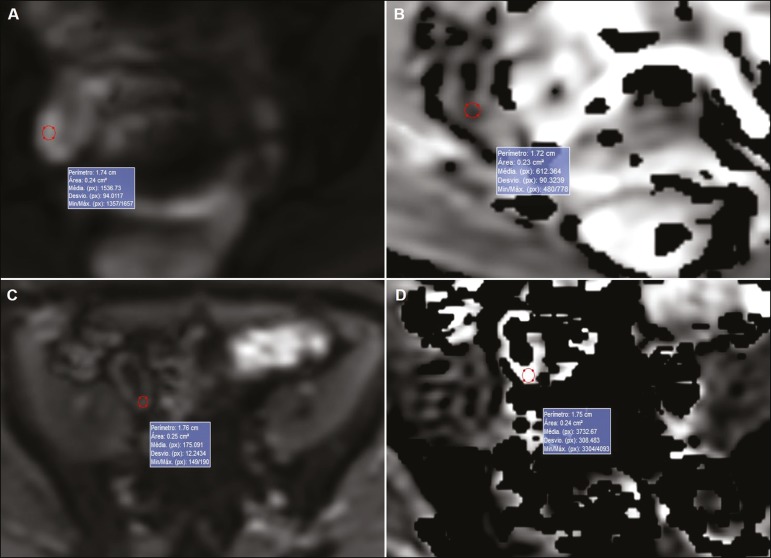
ADC measurements on MRE with ROIs in the intestinal wall. **A,B:** MRE of a 37-year-old female with active Crohn’s disease detected by colonoscopy who presented restricted diffusion, in **A**, and a low ADC value (0.612 × 10^−3^ mm^2^/s), in **B**. **C,D:** MRE of a 32-year-old female with inactive Crohn’s disease on colonoscopy who presented no restricted diffusion, in **C**, and a high ADC value (3.732 × 10^−3^ mm^2^/s), in **D**.

Figure 1 illustrates the MRE assessments of a patient with active inflammation detected by colonoscopy (Figures 1A and 1B) and of a patient with inactive disease (Figures 1C and 1D).

### Interobserver agreement

The interobserver agreement for restricted diffusion and for inflammation identified by colonoscopy was almost perfect (κ = 0.82; *p* < 0.001). The interobserver concordance was excellent for the MaRIA (ICC = 0.83; *p* < 0.001) and the Clermont score (ICC = 0.83; *p* < 0.001), whereas it was substantial for the ADC value (ICC = 0.73; *p* < 0.001) and poor for the RCE (ICC = 0.32; *p* = 0.124).

### Cut-off points

**Figure 2:**
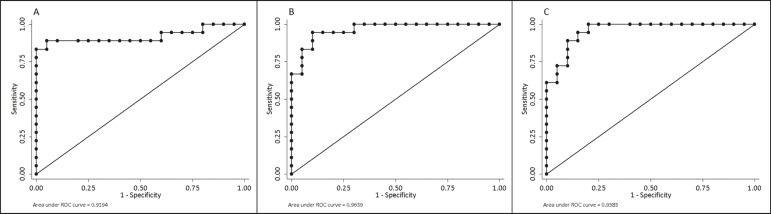
ROC curves illustrating the relationships that inflammation on colonoscopy had with ADC values, the MaRIA, and the Clermont score. **A:** ADC (AUC = 0.9194). **B:** MaRIA (AUC = 0.9639). **C:** Clermont score (AUC = 0.9583).

We evaluated the ROC curves for inflammation identified by colonoscopy in relation to that identified by continuous ADC values (AUC = 0.9194; Figure 2A). The diffusion sequences showed AUCs of 0.9639 and 0.9583 for the MaRIA (Figure 2B) and the Clermont score (Figure 2C), respectively. We found the best ADC cut-off point for detecting colonoscopy-confirmed inflammation to be 2.1 × 10^−3^ mm^2^/s, which had a sensitivity of 88.8% and a specificity of 95.0%.

### Correlation between inflammation identified by colonoscopy and the DWI sequence

**Figure 3:**
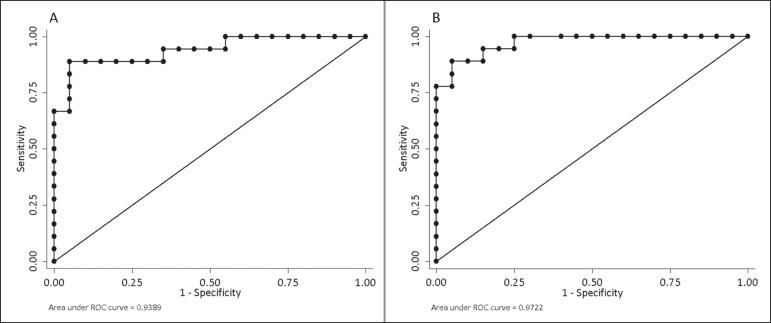
ROC curves illustrating the relationships that DWI had with the MaRIA and the Clermont score. **A:** MaRIA (AUC = 0.9389). **B:** Clermont score (AUC = 0.9722).

The DWI sequence presented an accuracy of 89.4%, sensitivity of 88.9%, and specificity of 90.0% for the detection of colonoscopy-confirmed inflammation. In addition, the DWI sequences were found to be predictive of the clinical scores, with AUCs of 0.9389 (95% CI: 0.8612-1.016) for the MaRIA (Figure 3A) and 0.9722 (95% CI: 0.9313-1.0131) for the Clermont score (Figure 3B).

## DISCUSSION

Recent studies have highlighted the importance of MRI in the improvement of the diagnosis of abdominal and gastrointestinal diseases^(13-17)^. Patients with active inflammation identified by colonoscopy had more significant restricted diffusion, more pronounced edema, and greater wall thickening than did those with inactive disease. The RCE did not differ significant between the two groups, suggesting that contrast enhancement alone is a nonspecific imaging finding, which can be related to inflammatory processes or other processes such as mucositis, graft-versus-host disease, intestinal contraction or subclinical distention, radiation enteritis, nonsteroidal anti-inflammatory drug-induced enteropathy, angioedema, vasculitis, and ischemia^(5)^.

Even the consensus recommendations for the evaluation, interpretation, and use of computed tomography and MRE in patients with Crohn’s disease of the small intestine state that contrast enhancement alone is nonspecific. However, the combination of the RCE and wall thickening has been shown to have moderately high sensitivity and specificity for detecting inflammation in the small intestine of patients with Crohn’s disease^(9,10,18-21)^. According to the recommendations^(10)^, edema and restricted diffusion are correlated with a finding of moderate to severe inflammation on colonoscopy. Although we did not categorize the degree of inflammation seen on colonoscopy in the present study, we observed more pronounced edema and greater restricted diffusion in the active inflammation group than in the inactive disease group. We found no statistically significant difference between the two groups in terms of stenosis, perhaps because inflammation and fibrosis can both promote stenosis^(22)^.

We found that the interobserver agreement was excellent for the evaluation of restricted diffusion, as well as being good for that of the MaRIA and Clermont score. There was no difference between the MaRIA and Clermont score in terms of the level of interobserver agreement. In contrast, Rimola et al.^(9)^ suggested that interobserver agreement was somewhat better for the MaRIA than for the Clermont score (ICC = 0.70 and 0.65, respectively). In the present study, the interobserver agreement for the ADC value was moderate. That is probably because it is often difficult to quantify the ADC in the small intestine because of the thickness of the wall (typically 1-2 mm) and the presence of intestinal peristalsis during image acquisition. Those limitations can be mitigated by shortening the image acquisition time. The interobserver concordance for the ADC value was negligible, which was in agreement with the results of previous studies^(4)^. In a prospective cohort study of 848 intestinal segments in 130 patients with Crohn’s disease, Buisson et al.^(4)^ demonstrated better interobserver agreement for ADC values than for RCE values, suggesting that the former are more reproducible for radiologists experienced in the use of quantitative parameters of DWI sequences.

For the patients with active inflammation, we found that the area under the curve for the ADC values showed good specificity and sensitivity at all of the cut-off points evaluated. An ADC cut-off value of 2.1 × 10^−3^ mm^2^/s was found to differentiate between normal and inflamed intestinal walls. Previous studies differentiating between the imaging findings in active inflammation and those obtained in inactive disease showed optimal ADC cut-off values of 1.9 × 10^−3^ mm^2^/s^(8)^ and 2.0 × 10^−3^ mm^2^/s^(6)^, similar to that identified in our study. The sensitivity and specificity reported in those studies-93.7% and 96.0%, respectively, reported by Hordonneau et al.^(8)^, and 84.0% and 91.0%, respectively, reported by Rimola et al. ^(9)^- differed slightly from those found in our study (88.8% and 95.0%, respectively). However, those studies involved a greater number of segments^(6,8)^.

Restricted diffusion on the ADC map acquired by the radiologist showed an accuracy of 89.4%, sensitivity of 88.9%, and specificity of 90.0% for detecting pathology-confirmed inflammation of the ileum. In addition, according to the recommendations of the most recent (2018) consensus^(10)^, MRE should be performed in patients with Crohn’s disease to detect inflammation of the small intestine and penetrating complications that are not seen on standard colonoscopy. In terms of restricted diffusion in relation to the MaRIA and the Clermont score, we found good accuracy among the tests.

Our study has some limitations. First, the patient sample was relatively small, and the study was conducted at a single center. Future studies with large samples are needed in order to validate the results presented here. In addition, we evaluated only the terminal ileum. Second, given the limitations inherent to the retrospective nature of this study, some clinical parameters could not be retrieved. Third, even with our best efforts, it was not possible to exclude the possibility of partial volume effects during the ADC assessment of the normal intestinal wall.

## CONCLUSION

Visual analysis of the DWI sequence has good accuracy in detecting inflamed ileal segments in patients with Crohn’s disease. Low ADC values have good sensitivity in detecting inflammation, which can also be detected by colonoscopy.
